# The efficacy of multi‐disciplinary lifestyle modifications in Taiwanese nonalcoholic steatohepatitis patients

**DOI:** 10.1002/kjm2.12833

**Published:** 2024-04-16

**Authors:** Ming‐Lun Yeh, Chia‐Yen Dai, Chung‐Feng Huang, Shiu‐Feng Huang, Pei‐Chien Tsai, Po‐Yau Hsu, Ching‐I Huang, Yu‐Ju Wei, Po‐Cheng Liang, Ming‐Jong Bair, Mei‐Hsuan Lee, Zu‐Yau Lin, Jee‐Fu Huang, Ming‐Lung Yu, Wan‐Long Chuang

**Affiliations:** ^1^ Hepatobiliary Division, Department of Internal Medicine Kaohsiung Medical University Hospital Kaohsiung Taiwan; ^2^ Graduate Institute of Clinical Medicine Kaohsiung Medical University Kaohsiung Taiwan; ^3^ Faculty of Internal Medicine, College of Medicine Kaohsiung Medical University Kaohsiung Taiwan; ^4^ Institute of Molecular and Genomic Medicine National Health Research Institutes Miaoli Taiwan; ^5^ Department of Anatomic Pathology Linko Chang Gung Memorial Hospital Taoyuan Taiwan; ^6^ Division of Gastroenterology, Department of Internal Medicine Taitung Mackay Memorial Hospital Taitung Taiwan; ^7^ Mackay Medical College New Taipei City Taiwan; ^8^ Institute of Clinical Medicine National Yang Ming Chiao Tung University Taipei Taiwan; ^9^ Division of Hepato‐Gastroenterology, Department of Internal Medicine, Kaohsiung Chang Gung Memorial Hospital National Sun Yat‐sen University Kaohsiung Taiwan; ^10^ School of Medicine, College of Medicine and Center of Excellence for Metabolic Associated Fatty Liver Disease National Sun Yat‐sen University Kaohsiung Taiwan

**Keywords:** fatty liver, lifestyle modification, NAFLD, NASH, steatohepatitis

## Abstract

Lifestyle modification is the standard of care for nonalcoholic fatty liver disease (NAFLD) patients. We aimed to investigate the efficacy of a short‐term lifestyle modification program in the disease course of Taiwanese nonalcoholic steatohepatitis (NASH) patients with paired biopsies. All patients received a 6‐month, strict multidisciplinary program of lifestyle modifications led by physicians, dieticians, and nursing staff. The histopathological and clinical features were assessed. The endpoints were normalization of transaminase levels, metabolic parameters, a decrease in the NAFLD activity score (NAS) ≥1, and a decrease in the fibrosis stage ≥1. We also aimed to elucidate the predictors associated with disease progression. A total of 37 patients with biopsy‐proven NASH were enrolled. The normalization of transaminase levels increased from 0% to 13.5%. There were also significantly increased proportions of patients with normal total cholesterol, triglyceride, and hemoglobin A1c levels. Fifteen (40.5%) patients had an increased NAS ≥1, whereas 10 (27.0%) patients had NAS regression. Twelve (32.4%) patients had increased fibrosis ≥1 stage. Only 2 (5.4%) patients experienced fibrosis regression. A high fasting plasma glucose (FPG) level was associated with NAS progression. Older age and higher transaminase and FPG levels were factors associated with fibrosis progression. Seven (18.9%) patients achieved a body weight reduction >3%, and 4 (57.1%) of them experienced NAS regression. No significant effect of weight reduction on the progression of fibrosis was observed. The short‐term lifestyle modification program significantly decreased liver enzymes and metabolic parameters in NASH patients. A more precise or intensive program may be needed for fibrosis improvement.

## 1 INTRODUCTION

Nonalcoholic fatty liver disease (NAFLD) has become the major cause of chronic liver disease worldwide. Metabolic liver disease is expected to be the leading cause of liver‐related morbidities, hepatocellular carcinoma (HCC), and liver transplantation in the near future.^1^, ^2^ The prevalence of NAFLD increased from 15% in 2010 to 25% in 2015.^3^ This scenario has been of particular importance in the past several decades throughout the Asia‐Pacific region in parallel with rapid Westernization.^4^, ^5^ NAFLD represents a spectrum of excessive hepatic fat accumulation, with insulin resistance (IR) as the main driver.^6^ Nonalcoholic steatohepatitis (NASH) is an extreme form of NAFLD in which necro‐inflammatory activities, such as hepatic ballooning and lobular inflammation, are observed.^7^

The disease course of complex metabolic liver disorders remains elusive. Generally, simple steatosis is considered to be a low risk factor for liver‐related outcomes. The risk of liver‐related events such as cirrhosis and HCC significantly increases once NASH is diagnosed.^8^ In addition, NASH patients have greater risks of mortality and liver‐related complications than patients with simple steatosis. A previous prospective paired biopsy study from Hong Kong demonstrated that 27% of NASH patients experienced fibrosis progression, whereas 25% of them experienced fibrosis regression during a follow‐up period of 36 months.^9^ A similar study from England showed that the spontaneous progression rate and regression rate were 42% and 18%, respectively, during a median follow‐up of 6.6 years.^10^ Our recent observation of NASH patients who underwent paired biopsies demonstrated that half of the patients experienced fibrosis progression, with an estimated annual fibrosis progression rate of 0.32/year during 20.5 months of follow‐up.^11^ Nonetheless, the transitional changes and/or disease course could be largely affected by many factors, such as sex, age, ethnicity, concomitant metabolic alterations, and lifestyle modifications. The clarification of the disease course is not only helpful in designing sufficient surveillance for high‐risk patients but also provides precise risk factors for preventive purposes.

Currently, no drug has been approved for NASH treatment. Lifestyle modifications remain an effective route for NASH improvement.^12^, ^13^ Previous studies demonstrated that a 52‐week lifestyle modification involving either a dietitian‐led program or vigorous weight reduction could lead to significant improvement or even resolution of NASH. Our recent study also showed that short‐term lifestyle modifications effectively improved hepatic steatosis as well as biochemical profiles in Taiwanese NAFLD patients.^14^ The efficacy of a short‐term sophisticated multidisciplinary lifestyle modification program for histological changes deserves exploration.

Consequently, we conducted a 24‐week lifestyle modification program to assess histopathological efficacy. The monthly visit intensive program was led by multidisciplinary teamwork. We aimed to assess the interaction between lifestyle modifications and disease progression in Taiwanese NASH patients. This study also aimed to elucidate the factors associated with disease progression and regression.

## 2 PATIENTS AND METHODS

This multicenter, prospective study enrolled biopsy‐proven NASH patients from a tertiary medical center and three core regional hospitals in southern Taiwan. The Kaohsiung Medical University Hospital Institutional Review Board approved the study. The study was conducted in compliance with the Declaration of Helsinki and the guidelines of the International Conference on Harmonization for Good Clinical Practice. Written informed consent was obtained from all the patients before enrollment.

### 2.1 Patient

Treatment‐naive patients aged 20–70 years who met all of the following inclusion criteria were considered eligible for this study: (1) had received a liver biopsy within 24 weeks of enrollment and had a histological diagnosis of NASH, (2) had an alanine aminotransferase (ALT) level of 1.3–5‐fold the upper limit of normal (ULN) for two occasions within 24 weeks of enrollment, and (3) had alcohol consumption of less than 20 g/day.

Patients who met any of the following criteria were excluded: (1) had a diagnosis of other liver diseases, such as viral hepatitis B or C, autoimmune hepatitis, primary biliary cholangitis, hemochromatosis, alpha‐1‐antitrypsin deficiency or Wilson's disease; (2) ALT or aspartate aminotransferase (AST) levels greater than 5‐fold of the ULN; (3) decompensated cirrhosis, overt hepatic failure, previous liver transplantation, or evidence of HCC; (4) concurrent use of drugs that cause hepatic steatosis or modify insulin resistance within 24 weeks of enrollment; (5) serum creatinine level >1.5‐fold of the ULN or calculated creatinine clearance as calculated by Cockcroft and Gault <60 mL/min; (6) metformin or insulin use within 24 weeks of enrollment or type 1 diabetes; or (7) seropositivity for hepatitis B surface antigen, hepatitis C antibody or human immunodeficiency virus antibody.

All patients were instructed by dietitians and/or hepatologists for standard lifestyle modification instructions during the study period. Compliance was measured monthly during the study visits. Patients underwent a second liver biopsy to evaluate the disease status of NASH after the completion of the 6‐month lifestyle modifications. The primary outcome of the current analysis was histopathological changes. The secondary outcome measurements included (1) normalization of transaminase levels and metabolic parameters, (2) the factors associated with disease progression and regression, and (3) the impact of body weight reduction on disease regression in NASH patients.

### 2.2 Lifestyle modifications

Lifestyle modifications aimed to reduce weight by 5%–10%. All participants were instructed by their physicians, nursing staff and/or dieticians monthly. The total daily caloric intake of each participant was calculated by the dietician according to their ideal body weight and daily activity index. The daily caloric intake was reduced sequentially by 500–1000 kcal per week until the daily basal metabolic rate or ideal body weight was reached. Participants were instructed to maintain a healthy diet that was high in protein and vegetables and to avoid sugars, sweeteners, processed meats, high‐fructose fruits, and refined foods. The food contents and amounts were reviewed by dieticians or nursing staff during their monthly visits. The physical activity control program emphasized and recommended moderate‐intensity activities or aerobic activities. Participants were encouraged to perform these activities at least 150 min a week, with at least 30 min a day and five times a week.

Enrolled patients routinely underwent blood tests, including fasting glucose (FPG), hemoglobin A1c (HbA1c), total cholesterol (T‐Chol), high‐density lipoprotein cholesterol (HDL‐C), low‐density lipoprotein cholesterol (LDL‐C), triglyceride (TG), gamma glutamyl transpeptidase (rGT), uric acid (UA), high‐sensitivity C‐reactive protein (hs‐CRP), AST and ALT levels, at enrollment and 6 months after enrollment. The homeostatic model assessment for insulin resistance (HOMA‐IR) score was calculated according to the following formula: FPG (mg/dL) × fasting insulin level (μU/mL)/405.

Liver biopsy was performed by experienced hepatologists with a 16‐ or 18‐gauge cutting needle, and liver tissue specimens of at least 2 cm in length were obtained. Liver tissue samples were stained with hematoxylin–eosin and reviewed by an independent hepato‐pathologist who was blinded to the patients. NASH was diagnosed according to the NASH Clinical Research Network.^15^ The NAFLD activity score (NAS) was graded from 0 to 8, including scores for steatosis (0–3), lobular inflammation (0–3) and hepatocellular ballooning (0–2). Fibrosis was staged from 0 to 4.

Metabolic syndrome (MetS) was defined as the presence of at least three of the following components: (1) waist circumference > 90 cm in males or >80 cm in females, (2) TG > 150 mg/dL, (3) HDL‐C < 40 mg/dL in males or <50 mg/dL in females, (4) blood pressure > 130/85 mmHg or current use of antihypertensive medications, and (5) FPG > 100 mg/dL or on oral antidiabetic agents or insulin.

### 2.3 Statistical analysis

Continuous variables are expressed as medians (interquartile ranges), and categorical variables are expressed as numbers and percentages. The Wilcoxon signed‐rank test, Mann–Whitney *U* test, chi‐square test and Fisher's exact test were used. All tests were two‐sided, and a *p* value <0.05 was considered to indicate statistical significance. All analyses were performed with the SPSS 20.0 statistical package (SPSS, Inc., Chicago, IL, USA).

## 3 RESULTS

**FIGURE 1:**
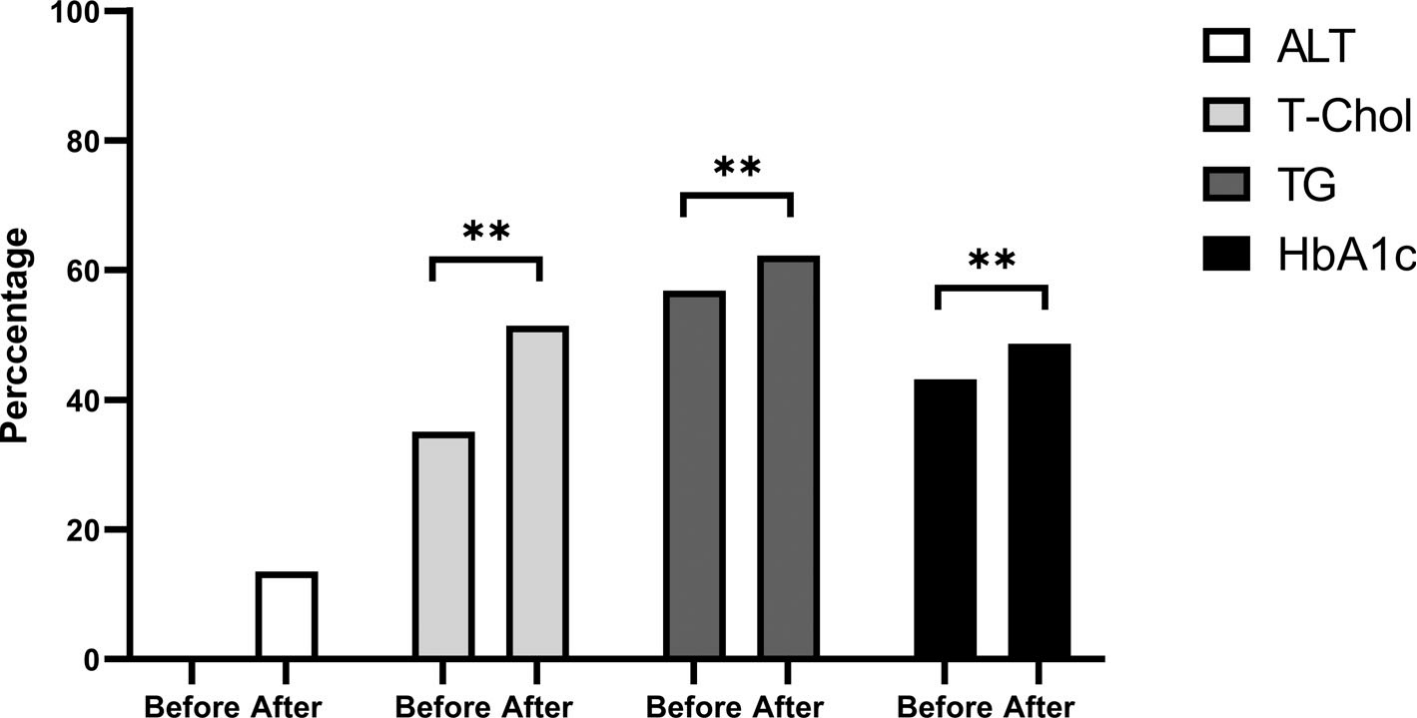
The proportion of patients with normal alanine aminotransferase, total cholesterol, triglyceride, and hemoglobin A1c levels before and after the 6‐month strict multidisciplinary program of lifestyle modifications. (** represents *p* < 0.01).

A total of 37 patients who met the inclusion/exclusion criteria and who underwent paired biopsy were enrolled in the analysis. The characteristics of all the patients before and after the 6‐month lifestyle modification program are shown in Table 1. There were significant decreases in AST, ALT, and TG levels from a median (interquartile range) of 45 (20), 75 (29) U/L, and 138 (95) mg/dL at baseline to 39 (23), 63 (38) U/L, and 130 (70) mg/dL at week 24 (*p* = 0.03, 0.05, and 0.03), respectively. In contrast, we did not observe significant decreases in T‐Chol, rGT, FPG, HbA1c, HDL‐C, LDL‐C, or UA levels. The proportion of patients with MetS decreased substantially from 67.6% (25/37) to 56.8% (21/37) (*p* = 0.08). There was no new‐onset diabetes or dyslipidemia. The ALT levels of five (13.5%) patients normalized at the end of the program. There were significant increases in the proportions of normalization in terms of T‐Chol (35.1%–51.4%, p < 0.01), TG (56.8% to 62.2%, *p* < 0.01), and HbA1c (43.2%–48.6%, *p* < 0.01) levels (Figure 1).

**TABLE 1:** Characteristics of all 37 patients before and after the 6‐month strict multidisciplinary lifestyle modification program.

	Before	After	*p*
Age, years	43 (22)		
Sex	32 (86.5)		
Body mass index	29.2 (4.7)	29.3 (5.7)	0.27
Hip circumference, cm	105 (14)	107 (12)	0.73
Waist circumference, cm	97 (12)	98 (14)	0.85
Hip/waist circumference ratio	1.09 (0.06)	1.10 (0.11)	0.98
Diabetes	10 (27.0)	10 (27.0)	
Hypertension	8 (21.6)	8 (21.6)	
Hyperlipidemia	19 (51.4)	19 (51.4)	
Metabolic syndrome	25 (67.6)	21 (56.8)	0.08
Platelet, ×10^3^/μL	239 (67)	234 (58)	0.23
AST, U/L	45 (20)	39 (23)	0.03
ALT, U/L	75 (29)	63 (38)	0.05
GGT, U/L	51 (39)	44 (43)	0.36
Creatinine, mg/dL	0.9 (0.3)	0.9 (0.3)	0.86
hs‐CRP, mg/L	0.2 (0.3)	0.2 (0.3)	0.72
Fasting glucose, mg/dL	100 (17)	105 (30)	0.68
Hemoglobin A1c, %	6.0 (0.8)	6.0 (1.0)	0.81
HOMAR‐IR	2.2 (2.3)	2.8 (3.0)	0.64
Total CHOL, mg/dL	208 (47)	197 (60)	0.09
HDL CHOL, mg/dL	43 (11)	42 (8)	0.79
LDL CHOL, mg/dL	131 (42)	132 (56)	0.32
Triglycerides, mg/dL	138 (95)	130 (70)	0.03
Uric acid, mg/dL	6.9 (2.3)	7.0 (1.8)	0.90

*Note*: Continuous data are presented as medians (IQRs); categorical data are presented as numbers (percentages); and statistical analysis was performed with the Wilcoxon signed‐rank test, chi‐square test and Fisher's exact test.

### 3.1 Progression of nash and fibrosis

**FIGURE 2:**
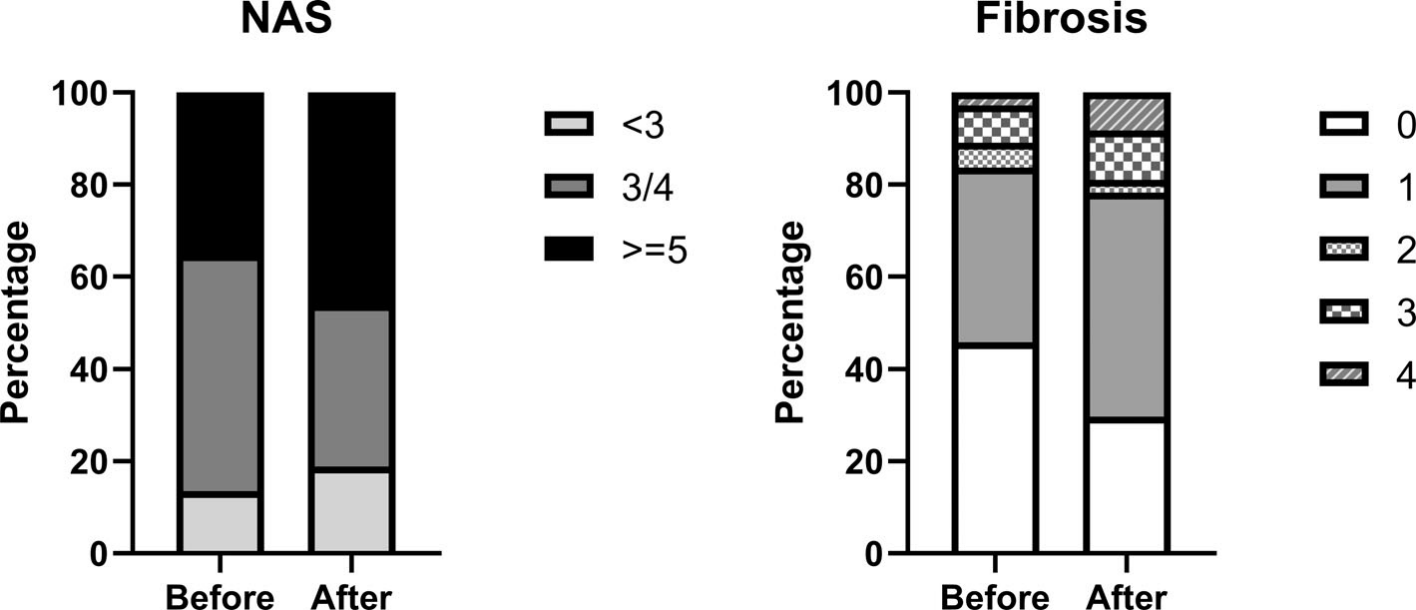
Distribution of NAFLD activity score (NAS) and fibrosis stage before and after the 6‐month strict multidisciplinary lifestyle modification program.

There were 15 (40.5%) patients who experienced NAS progression and 15 (27.0%) who experienced regression of at least one score. Of the 15 patients who experienced NAS progression, 10 had increased steatosis of one grade. Five patients had increased inflammation of one grade. Six patients had increased ballooning of one grade. Only two patients had simultaneously increased steatosis, inflammation, and ballooning for one grade. Body weight gain might explain the progression of NAS. Twelve (80%) of the 15 patients with NAS progression had increased body weight, which was a significantly greater proportion than that of the patients without NAS progression (5 [22.7%] out of 22). Five (13.5%) and seven (18.9%) patients had NAS <3 before and after the program, respectively. The number of patients with definite NASH (NAS ≥5) increased from 13 (35.1%) patients to 17 (45.9%) patients after the program (*p* < 0.01) (Figure 2).

For liver fibrosis, 12 (32.4%) patients had fibrosis that progressed ≥1 stage, whereas only 2 (5.4%) patients had regression of fibrosis. The proportion of patients with fibrosis ≥stage 2 increased from 16.2% to 21.6% (Figure 2). The two patients who experienced fibrosis regression were males and had BMIs of 26.8 and 27.2 kg/m^2^, respectively, at enrollment. The NAS also decreased from 4 to 3 and 2 months after the 6‐month lifestyle modification program, respectively. One patient experienced a body weight reduction of 3.6%, and the other patient lost 1.7% of weight. These patients exhibited significant improvements in liver enzymes and triglyceride levels.

### 3.2 Factors associated with the progression of nash and fibrosis

We then analyzed the risk factors associated with fibrosis progression and found that older age and higher AST and FPG levels were the factors associated with fibrosis progression. There were significant differences in the changes in AST, ALT, HbA1c, and T‐Chol levels between patients with and without fibrosis progression. (Table 2).

**TABLE 2:** Factors associated with fibrosis progression after the 6‐month strict multidisciplinary program of lifestyle modifications.

Factors at 1st biopsy	Progression, *n* = 12	Nonprogression, *n* = 25	*p*
Age	51 (11)	36 (17)	<0.01
Sex	10 (83.3)	22 (88.0)	1.00
Body mass index	29.0 (3.7)	30.6 (5.2)	0.15
Hip circumference	104 (5)	110 (15)	0.05
Waist circumference	94 (14)	99 (14)	0.19
Hip/waist circumference ratio	1.08 (0.04)	1.10 (0.06)	0.40
Diabetes	5 (41.7)	5 (20.0)	0.24
Hypertension	4 (33.3)	4 (16.0)	0.39
Hyperlipidemia	6 (50.0)	13 (52.0)	1.00
Metabolic syndrome	9 (75.0)	16 (64.0)	0.71
Platelet	216 (69)	261 (57)	0.10
AST	53 (35)	41 (20)	0.03
ALT	82 (51)	71 (28)	0.11
GGT	48 (37)	53 (39)	0.85
Creatinine	1.0 (0.3)	0.9 (0.3)	0.42
hs‐CRP	0.2 (0.3)	0.2 (0.5)	0.90
Fasting glucose	111 (37)	100 (16)	0.03
Hemoglobin A1c, %	6.3 (1.0)	5.8 (0.9)	0.06
HOMAR‐IR	2.6 (2.3)	2.1 (2.0)	0.37
Total CHOL	219 (27)	200 (52)	0.08
HDL CHOL	43 (10)	42 (12)	0.71
LDL CHOL	138 (31)	124 (46)	0.29
Triglycerides	155 (120)	137 (80)	0.26
Uric acid	6.8 (3.1)	6.9 (2)	0.72

*Note*: Continuous data are presented as medians (IQRs); categorical data are presented as numbers (percentages); and statistical data were analyzed with the Mann–Whitney *U* test, chi–square test and Fisher's exact test.

We also analyzed the factors associated with NAS progression or regression. Higher rGT and FPG levels were associated with NAS progression. Patients who experienced NAS progression had greater BMIs, hip/waist circumferences, and AST and ALT levels after the program than did those without NAS progression. A higher creatinine level at enrollment and an increased platelet count after the program were predictors of NAS regression (Table 3).

**TABLE 3:** Factors associated with NAS progression or regression after the 6‐month strict multidisciplinary program of lifestyle modifications.

Factors at first biopsy	Progression, *n* = 15	Nonprogression, *n* = 22	*p*	Factors at first biopsy	Regression, *n* = 10	Nonregression, *n* = 27	*p*
Age	37 (27)	46 (20)	0.43	Age	49 (17)	38 (21)	0.12
Male sex	14 (93.3)	18 (81.8)	0.63	Male sex	10 (100)	22 (81.5)	0.30
Body mass index	30.4 (4.6)	29.2 (4.6)	0.60	Body mass index	28.2 (5.8)	29.8 (4.6)	0.25
Hip circumference	109 (18)	105 (9)	0.70	Hip circumference	104 (8)	109 (14)	0.26
Waist circumference	98 (15)	97 (11)	0.95	Waist circumference	96 (12)	99 (13)	0.87
Hip/waist circumference ratio	1.10 (0.08)	1.08 (0.05)	0.28	Hip/waist circumference ratio	1.08 (0.03)	1.10 (0.07)	0.07
Diabetes	6 (40.0)	4 (18.2)	0.26	Diabetes	2 (20.0)	8 (29.6)	0.69
Hypertension	5 (33.3)	3 (13.6)	0.23	Hypertension	0 (0)	8 (29.6)	0.08
Hyperlipidemia	8 (53.3)	11 (50.0)	1.00	Hyperlipidemia	6 (60.0)	13 (48.1)	0.71
Metabolic syndrome	12 (80.0)	13 (59.1)	0.29	Metabolic syndrome	5 (50.0)	20 (74.1)	0.24
Platelet	261 (92)	237 (50)	0.90	Platelet	235 (61)	261 (73)	0.76
AST	41 (18)	52 (26)	0.20	AST	47 (21)	45 (17)	0.46
ALT	72 (17)	82 (43)	0.32	ALT	65 (32)	75 (27)	0.13
GGT	63 (39)	44 (34)	0.04	GGT	38 (31)	52 (32)	0.11
Creatinine	0.9 (0.3)	0.9 (0.3)	0.49	Creatinine	1.0 (0.2)	0.8 (0.3)	0.01
hs‐CRP	0.2 (0.4)	0.2 (0.3)	0.84	hs‐CRP	0.2 (0.4)	0.2 (0.3)	0.53
Fasting glucose	109 (41)	99 (16)	0.02	Fasting glucose	99 (15)	108 (26)	0.31
Hemoglobin A1c	6.3 (1.9)	6.0 (0.9)	0.07	Hemoglobin A1c	6.1 (0.5)	6.0 (1.0)	0.89
HOMAR‐IR	2.3 (2.4)	2.1 (2.3)	0.46	HOMAR‐IR	1.7 (2.0)	2.3 (2.5)	0.11
Total CHOL	201 (49)	218 (53)	0.44	Total CHOL	213 (50)	204 (51)	0.71
HDL CHOL	37 (11)	43 (10)	0.13	HDL CHOL	45 (8)	42 (10)	0.47
LDL CHOL	127 (37)	134 (49)	0.60	LDL CHOL	135 (45)	127 (49)	0.68
Triglycerides	154 (124)	138 (88)	0.76	Triglycerides	147 (100)	138 (108)	0.85
Uric acid	6.9 (3.4)	6.5 (2.1)	0.33	Uric acid	7.1 (1.5)	6.6 (2.5)	0.66

*Note*: Continuous data are presented as medians (IQRs); categorical data are presented as numbers (percentages); and statistical data were analyzed with the Mann–Whitney *U* test, chi‐square test and Fisher's exact test.

### 3.3 Effect of body weight reduction on the progression of nash and fibrosis

To investigate the effect of body weight reduction on the severity of NASH and fibrosis, we divided patients according to body weight reduction by 3% or 5%. Seven (18.9%) and five (13.5%) patients experienced a body weight reduction of more than 3% and 5%, respectively. As shown in Table 4, patients with a body weight reduction ≥3% had a substantially greater percentage of patients with a decrease in the NAS ≥1 than did their counterparts (57.1% vs. 20.0%, *p* = 0.07). Moreover, none of the 7 patients with a weight reduction ≥3% experienced an increase in the NAS, but 15 (50%) of the 30 patients with a weight reduction <3% experienced an increase in the NAS. Similar results were also observed in patients with a body weight reduction of 5%. We also observed that body weight reduction improved hepatic steatosis. We did not observe any effect of weight reduction on the progression of hepatic fibrosis.

**TABLE 4:** Comparison of liver histology between patients with and without body weight reduction of 3% and 5% after the 6‐month strict multidisciplinary program of lifestyle modifications.

	Weight reduction					
	≥3%	<3%	*p*	≥5%	<5%	*p*
	*n* = 7	*n* = 30		*n* = 5	*n* = 32	
NAS increases for one score	0 (0)	15 (50.0)	0.03	0 (0)	15 (46.9)	0.07
NAS decreases for one score	4 (57.1)	6 (20.0)	0.07	2 (40.0)	8 (25.0)	0.60
Fibrosis increases one stage	3 (42.9)	9 (30.0)	0.66	2 (40.0)	10 (31.3)	1.00
Fibrosis decreases one stage	1 (14.3)	1 (3.3)	0.35	0 (0)	2 (6.3)	1.00

## 4 DISCUSSION

NAFLD is the most common liver disorder globally, with a prevalence of 25%.^16^ Its incidence has rapidly progressed in the past several decades throughout the Asia‐Pacific region in parallel with the rapid Westernization of the region.^17^ Despite having a significantly lower BMI and lower rate of obesity than other ethnic groups, Asians have a significant prevalence of NAFLD as well as other metabolic disorders, such as hypertension, T2DM, and MetS.^18^ However, the disease course of this complex metabolic liver disease remains elusive. The optimal management also awaits investigation in a clinical setting. We investigated the efficacy of a 6‐month lifestyle modification program for the disease course of Asian NASH patients. The study demonstrated that NAS progression and regression were observed in 40.5% and 27.0% of patients, respectively. The 6‐month lifestyle modifications achieved a body weight reduction >3% in less than 20% of the patients. A total of 57.1% of patients experienced NAS regression when their body weight decreased >3% during the study period. In addition, only two (5.4%) patients experienced fibrosis regression, whereas one‐third of the patients experienced fibrosis progression for more than one stage during the study period. Our results showed the effect of lifestyle modification program‐induced body weight reduction on the prevention of NASH but not fibrosis progression, suggesting the modest efficacy of lifestyle modifications within half a year.

Liver fibrosis is the process of the formation and deposition of fibrous connective tissue and extracellular matrix, leading to progressive structural tissue remodeling. It is the sequela of necroinflammation and/or cellular insults and is also a major determinant and a significant predictor of long‐term outcomes in NAFLD patients. There is a dose‐dependent association between the risk of mortality and the stage of fibrosis, in which a higher risk of mortality is associated with a higher stage of fibrosis.^7^, ^19^ One recent meta‐analysis of five large‐scale studies demonstrated that NASH patients with advanced fibrosis had a significantly greater risk of all‐cause mortality and liver‐related mortality than did NASH patients without fibrosis. Moreover, the risk of liver‐related mortality increases on an exponential rather than linear scale with increasing fibrosis stage.^20^ The current study demonstrated that one‐third of the patients experienced fibrosis progression during the short study period. Notably, all the patients were under strict multidisciplinary efforts, and they received monthly education for lifestyle modifications. These results suggest that NASH is a progressive liver disease that may carry the risk for all‐cause mortality and liver‐related mortality upon continuous fibrogenesis. Our study provides evidence for determining the speed of disease progression. This may raise concerns about optimal surveillance strategies, at least in Taiwanese NASH patients.^21^

NAFLD has two histological subtypes: NAFL (simple steatosis with/without mild inflammation) in 70%–75% of patients and NASH (steatohepatitis with/without fibrosis) in 25%–30% of patients.^22^ Regarding the fibrosis progression of NAFLD, one systemic review included 411 biopsy‐proven NAFLD patients from 11 cohort studies.^23^ They reported a 33.6% rate of progression and 22.3% rate of regression. NASH patients had a higher annual fibrosis progression rate of 0.14 stages, which corresponded to 1 stage of progression over 7.1 years. Another recent study involving 36 NAFLD patients (26 with NAFL and 10 with NASH) with paired liver biopsies reported that 27% of patients with NAFL progressed to NASH during a median 3.8 years of follow‐up.^24^ Fibrosis progressed in 15 (42%) patients and regressed in 9 (25%) patients. Interestingly, more than 50% of patients with NASH no longer met the criteria for NASH according to the second biopsy. naturally. Our prior small cohort study showed similar results for the progression of NASH and fibrosis in 60% and 50% of patients, respectively, during a 20‐month follow‐up.^11^ All the data suggest that NAFLD is a progressive disease with dynamic changes in its natural course. Unlike prior studies, we presented the dynamics of NASH and fibrosis after a strict, short‐term lifestyle intervention. Unexpectedly, the results demonstrated that NASH progressed in as many as 40% of patients, and only a small proportion of patients experienced fibrosis regression. Our data suggested that short‐term lifestyle modification alone might not be sufficient to control NASH, especially hepatic fibrosis. Interventions other than lifestyle modification or a more extensive and vigorous program with a longer period are therefore mandatory for NASH control.

A greater extent of body weight reduction improved the severity of NASH. According to a prior study of 261 patients who underwent paired biopsy, a body weight reduction ≥5% achieved 58% NASH resolution and 82% 2‐point NAS reduction through a 52‐week lifestyle modification.^13^ Fibrosis even regressed in 45% of patients who lost ≥10% of their body weight. In our study, only 19.4% and 13.9% of patients had weight reductions ≥3% and ≥5%, respectively, which were lower than the 30% weight reductions ≥5% reported in a prior study. This was because of the shorter duration of intervention in our study. Similarly, we also found that 57% of patients with a weight reduction ≥3% had at least a one‐point reduction in the NAS. However, a weight reduction ≥3% may not be enough to prevent fibrosis progression, so 43% of patients had at least one stage of fibrosis progression. Our results echo those of a prior study showing that a greater reduction in body weight was necessary for NASH resolution and fibrosis regression.

One limitation of the present study was the relatively small sample size. Liver biopsy is an invasive procedure with the possibility of major complications. Therefore, it is difficult to encourage patients with chronic liver disease to undergo liver biopsy, especially given recent advances in noninvasive assessments of hepatic fibrosis and steatosis. Moreover, the necessity of a second liver biopsy also increases the difficulty of patient enrollment. Another limitation was that we were unable to ensure the adherence of patients to lifestyle interventions. According to the study design, the enrolled patients were closely monitored for standard lifestyle modification instructions throughout the entire study period. However, we were not sure that all the patients were completely adherent when they were discharged from the clinic. This might explain why only one‐fifth of patients experienced weight reduction of more than 3%.

In conclusion, our study demonstrated that a short‐term lifestyle modification program significantly ameliorated hepatic steatosis and achieved normalization of liver and metabolic parameters in NASH patients. The modest efficacy in the prevention of NAS progression among those who experienced body weight reduction suggested that a more precise or long‐term program may be needed. In addition, the specific surveillance program for liver‐related outcomes deserves further investigation and validation due to the high incidence of fibrosis progression in Taiwanese NASH patients.

## CONFLICT OF INTEREST STATEMENT

All authors declare no conflict of interest.

## ETHICS APPROVAL

The Institutional Review Board of the Kaohsiung Medical University Hospital approved the study (KMUHIRB‐G(II)‐20170013). The study was conducted in compliance with the Declaration of Helsinki and the Good Clinical Practice guidelines of the International Conference on Harmonization.
